# *In vivo *multiplex quantitative analysis of 3 forms of alpha melanocyte stimulating hormone in pituitary of prolyl endopeptidase deficient mice

**DOI:** 10.1186/1756-6606-2-14

**Published:** 2009-06-02

**Authors:** Bertrand Perroud, Rudy J Alvarado, Glenda M Espinal, Alex R Morado, Brett S Phinney, Craig H Warden

**Affiliations:** 1 Genome Center and Bioinformatics Program, University of California, Davis, California, USA; 2 Genome Center Proteomics Core, University of California, Davis, California, USA; 3 Rowe program in Genetics, University of California, Davis, California, USA; 4 Section of Neurobiology, Physiology and Behavior, Department of Pediatrics, University of California, Davis, California, USA

## Abstract

**Background:**

*In vitro *reactions are useful to identify putative enzyme substrates, but *in vivo *validation is required to identify actual enzyme substrates that have biological meaning. To investigate *in vivo *effects of prolyl endopeptidase (PREP), a serine protease, on alpha melanocyte stimulating hormone (α-MSH), we developed a new mass spectrometry based technique to quantitate, in multiplex, the various forms of α-MSH.

**Methods:**

Using Multiple Reaction Monitoring (MRM), we analyzed peptide transitions to quantify three different forms of α-MSH. Transitions were first confirmed using standard peptides. Samples were then analyzed by mass spectrometry using a triple quadrupole mass spectrometer, after elution from a reverse phase C18 column by a gradient of acetonitrile.

**Results:**

We first demonstrate *in vitro *that PREP digests biological active alpha melanocyte stimulating hormone (α-MSH_1–13_), by cleaving the terminal amidated valine and releasing a truncated alpha melanocyte stimulating hormone (α-MSH_1–12_) product – the 12 residues α-MSH form. We then use the technique *in vivo *to analyze the MRM transitions of the three different forms of α-MSH: the deacetylated α-MSH_1–13_, the acetylated α-MSH_1–13 _and the truncated form α-MSH_1–12_. For this experiment, we used a mouse model (PREP-GT) in which the serine protease, prolyl endopeptidase, is deficient due to a genetrap insertion. Here we report that the ratio between acetylated α-MSH_1–13 _and α-MSH_1–12 _is significantly increased (P-value = 0.015, N = 6) in the pituitaries of PREP-GT mice when compared to wild type littermates. In addition no significant changes were revealed in the relative level of α-MSH_1–13 _versus the deacetylated α-MSH_1–13_. These results combined with the demonstration that PREP digests α-MSH_1–13_* in vitro*, strongly suggest that α-MSH_1–13 _is an *in vivo *substrate of PREP.

**Conclusion:**

The multiplex targeted quantitative peptidomics technique we present in this study will be decidedly useful to monitor several neuropeptide enzymatic reactions *in vivo *under varying conditions.

## Introduction

*In vitro *reactions have been broadly applied to study the characteristics of enzymatic reactions. However results from *in vitro *experiments may not reflect true biologic reactions. The enzymatic reactions revealed *in vitro *may not even have the possibility of occurring *in vivo *because of substrate availability due to of space, timing or other restrictions. Therefore it is critical to validate enzymatic reactions *in vivo*. Verifications to ensure co-localization of the substrate and enzyme include immunohistochemistry or in situ staining techniques, like X-Gal staining. Additionally, specific inhibitors have also been used to demonstrate effect on enzyme activity, substrate or product levels. However, specificity of the inhibitor is frequently an issue. It has been difficult to demonstrate a direct effect between a specific substrate and enzyme. Techniques based on radioimmunoassay (RIA) are used to quantify specific targets from a reaction but they often lack the specificity required to distinguish between various biologically important forms of the targeted molecules. Therefore quantifying specific molecules has been a major hurdle to decipher important biological reactions. Another important issue is proper normalization when processing multiple biological samples and in particular for signaling neuropeptides with varying concentration. Reliable normalization can be achieved by selecting of endogenous controls from the targeted data set, using statistical treatment [1]. In the case of enzymatic reactions, the optimal normalization is to measure the ratio of enzyme substrate to enzyme product.

In recent years, several studies have demonstrated the suitability of mass spectrometry (MS) quantitative peptidomics, using stable isotope labels, particularly on neuropeptides from mice hypothalamus and pituitary (for review [2]). More recently, targeted quantitative proteomics methods using triple quadrupole MS instruments, allowed for quantitation without the use of stable isotope labels [3]. This new method is highly sensitive because the MS instrument is focused on specific targets, instead of performing wide scans.

Neuropeptide hormones involve multiple maturation steps, including cleavage by proteases, multiple post-translational modifications and deactivation or degradation processes. Further, rapid perturbations of the neuropeptide hormone concentration are a confounding factor in quantitation analysis. Here, we propose a new MS technique based on Multiple Reaction Monitoring (MRM) transitions to quantitate *in vivo *the various forms of the neuropeptide α-MSH, in a multiplex fashion.

Prohormone proopiomelanocortin (POMC) is the precursor of α-MSH, an important anorexigenic neuromodulator and immunomodulator. α-MSH is mainly produced in the pituitary and hypothalamus from the cleavage of POMC by successive action of prohormone convertases 1 and 2 and carboxypeptidase E. α-MSH then goes through several post-translational modifications to become anorexigenically active. These post-translational modifications include C terminus amidation by α-amidating monooxygenase and N terminus acetylation by an N-acetyltransferase (for review [4]). The processing of POMC in several endopeptides, occurs in the central nervous system, but also in peripheral organ such as skin, where α-MSH is indeed involved in the stimulation of melanocytes, but also in cutaneous stress responses [5]. There are multiple melanocortin receptors, involved in several functions. α-MSH and derived peptides such as the tripeptide KPV, are involved in anti-inflammatory mechanisms through melanocortin receptors 4 (MC4R) and possibly 1 (MC1R) [6] (for review [7]). α-MSH_1–13 _has been shown to bind to hypothalamus melanocortin receptors 3 and 4 (MC3R and MC4R) neurons and is also associated to reduce food intake (for review [8]).

Mutations in the melanocortin pathway are the most common known causes of mendelian forms of human obesity [9]. Additionally, studies of mouse models have identified candidate obesity genes that influence the melanocortin pathway. Congenic mouse strains are identical to a background strain except for a defined chromosomal region from a donor strain. Obesity differences between congenic and background are due to donor strain alleles in the congenic region. Several studies have identified congenic mice in which the donor chromosomal region contains genes that may influence levels of melanocortin hormones, such as prohormone convertase subtilisin/kexin-2 (Pcsk2) [10] and secretogranin V (Scg5) [11]. However, assessment of the *in vivo *effects of these genes on melanocortin pathways has been limited to studies of one or a few peptide hormones at a time [11], or has been limited in ability to study all forms of melanocortins by the availability of antibodies. Other studies have shown effects of mutations in the melanocortin pathway on responses to diet and exercise, but were unable to determine specific changes in melanocortin hormones due to lack of general assays to quantitate multiple peptide hormones in individual samples [12].

The mouse model used in this study, PREP-GT, is deficient in the serine protease, Prolyl endopeptidase (PREP, also known as prolyl oligopeptidase – POP), due to a genetrap insertion [13]. PREP is a post-proline cleaving enzyme (for review [14]), which is expressed in a wide variety of tissues, brain, lung, kidney, heart, muscle, uterus, spleen, macrophages and others [15,16]. It has been shown to digest *in vitro *α-MSH [17]. However, the product of the enzymatic reaction was not characterized. PREP has also many other putative substrates (for review [16]). Serum PREP levels are correlated with depression, mania and anorexia [18,19]. PREP has been associated with Alzheimer's disease and neurodegeneration, implicating PREP activity in memory [20]. No previous studies suggested that PREP levels influence obesity. However, the mouse model (PREP-GT), used in this study has shown a maternal influence of PREP on the fat mass of adult progeny. This mouse model has been phenotypically characterized and it exhibits a parent of origin effects on obesity [13].

Here we measure three forms of α-MSH from mouse pituitary: deacetylated α-MSH_1–13_, acetylated α-MSH_1–13 _and α-MSH_1–12_, a truncated form which results from the activity of serine proteases, in PREP-GT mice and demonstrate a highly sensitive and specific method to quantitate neuropeptides *in vivo*.

## Results

### α-MSH_1–12 _is the product of PREP activity on the substrate α-MSH_1–13_

**Figure 1:**
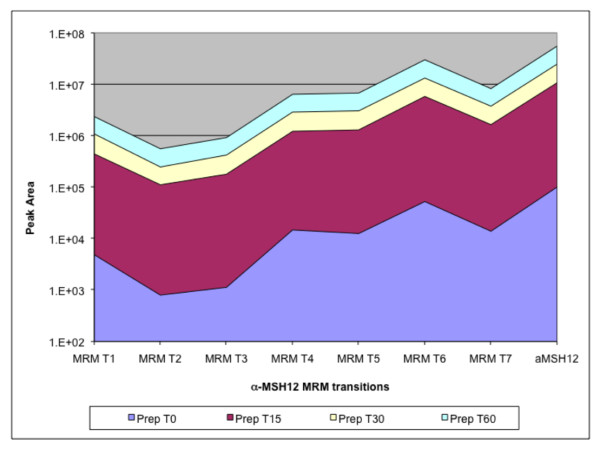
**The seven α-MSH_1–12 _MRM transitions (MRMT_1 to MRMT_7) used are increasing in a synchronous fashion during the time course**. Peak areas of each MRM transition are shown for each time point on a log scale.

**Figure 2:**
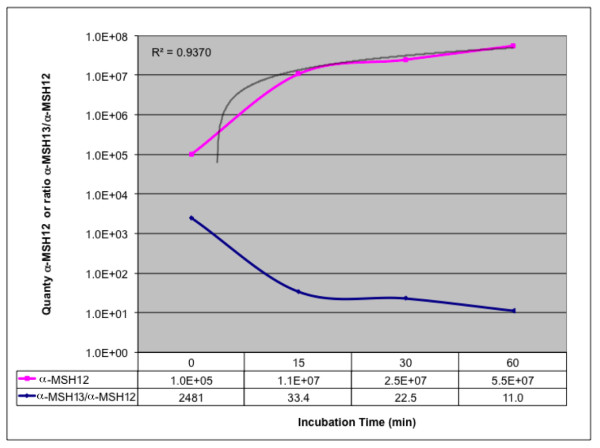
***In vitro *time course of PREP activity on α-MSH_1–13_**. The increase of α-MSH_1–12 _(pink line) fits a linear regression with R^2 ^= 0.9370 (black line). The ratio of α-MSH_1–13_/α-MSH_1–12 _decreases in a corresponding fashion (blue line).

We first tested the ability of multiplex, targeted, quantitative peptidomics by analyzing the *in vitro *reaction of PREP on α-MSH_1–13_. We identified 5 and 7 MRM transitions using standard α-MSH_1–13 _and α-MSH_1–12_, respectively. Then these MRM transitions were targeted on a time course experiment, in which we exposed α-MSH_1–13 _to recombinant PREP (rPREP). We observed a very good concordance of the α-MSH_1–12 _quantitation for each transition (Fig. 1), demonstrated by the parallel plots of the four time points. The increase in the quantity of α-MSH_1–12 _over time demonstrates that the enzymatic activity of rPREP on α-MSH_1–13_produced α-MSH_1–12 _[see Additional File 1]. α-MSH_1–12 _is the product rPREP activity, we used it to normalize the quantitative results obtained in the five MRM transitions of α-MSH_1–13_. Using this method, we were able to confirm the relative amount of α-MSH_1–13 _decreases over time in the reaction mixes (Fig. 2) This experiment demonstrated that α-MSH_1–13 _is an *in vitro *substrate of rPREP and the product of the enzymatic reaction is α-MSH_1–12_. It also confirmed the suitability of the MRM transitions we identified to quantitate both α-MSH_1–13 _and α-MSH_1–12 _in multiplex.

### PREP-GT pituitary multiplex quantitative analysis of three forms of α-MSH

**Figure 3:**
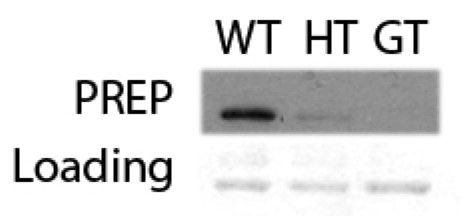
**Western blots of pituitary from PREP-GT littermate mice with PREP antibody: From left to right Wild Type (WT), PREP-GT heterozygotes (HT) and PREP-GT homozygotes (GT)**. The loading is shown below from a Ponceau S stained gel.

We developped an MS method with 4, 5 and 7 MRM transitions, respectively, to quantitate *in vivo *the deacetylated α-MSH_1–13_, the anorexigenically active acetylated α-MSH_1–13 _and the truncated form, α-MSH_1–12_, which results from the activity of PREP [see Additional File 2]. The pituitary PREP level in the PREP-GT mice were checked by western blotting and these mice are clearly deficient in PREP enzyme in the homozygote mice (Fig. 3).

**Figure 4:**
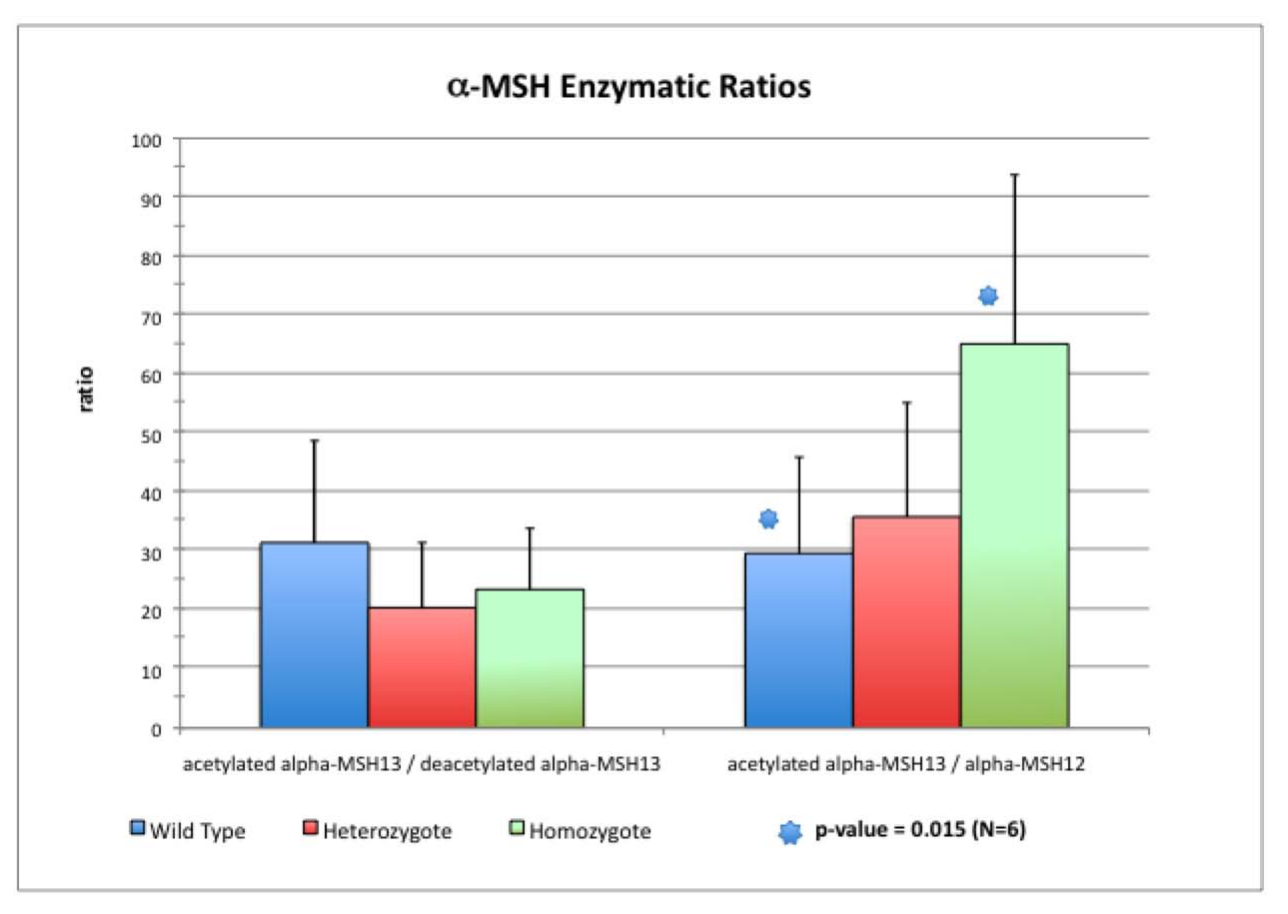
**ratio of acetylated α-MSH_1–13 _acetylated over deacetylated α-MSH_1–13 _and acetylated α-MSH_1–13 _over α-MSH_1–12 _in three genotypes of littermate mice from PREP-GT mouse strain**. Left: acetylated α-MSH_1–13_/deacetylated α-MSH_1–13_; Right: α-MSH_1–13_/α-MSH_1–12_.

In this experiment, the three forms of α-MSH were measured in pituitaries of littermates generated from parents heterozygous for the PREP-GT knockout (*PREP*^+/*gt*^). The levels of deacetylated α MSH_1–13 _and acetylated α MSH_1–13 _were not significantly different between the three classes of littermates [homozygous PREP-GT (*PREP*^*gt*/*gt*^), heterozygous (*PREP*^+/*gt*^), and homozygous wild type (*PREP*^+/+^)]. However, there was a statistically significant increase in α-MSH_1–13_, level relative to α-MSH_1–12 _level, in *PREP*^*gt*/*gt *^mice (Fig. 4). The fold change of acetylated α-MSH_1–13 _vs acetylated α-MSH_1–12_, between the *PREP*^*gt*/*gt *^mice and the wild type littermates was 2.2, with a one tail Student ttest p-value of 0.015 (N = 6).

## Discussion

We demonstrated a label free multiplex quantitative peptidomics method that allows the targeting of multiple peptides and their isoforms. A key feature is the multiplex ability that allows the comparison of several peptide targets in a single sample. This is very advantageous when monitoring an enzyme substrate and its product and therefore allows targeting of specific enzymatic activities. This method differs from current quantitative MS techniques using isotopically labeled tags. Typically in these techniques, an isotopically labeled tag is attached to the peptides in one sample and then mixed with another sample, which were labeled with a chemically identical tag that differs in mass, generally due to different numbers of heavy isotopes of carbon, hydrogen or nitrogen. Because the tags are chemically identical but differ in mass, one can compare the intensity of the labeled peptide pairs to determine relative abundances of the peptides in the sample. This is a great technique for discovery of differential peptides. However it is limited by the characteristics of the labeling which can introduce a label bias. Indeed, an isotopic label needs a site to attach itself on the target molecules, which creates a bias for some peptides. For example, TMAB label, along with many other tags, requires a free amine [2] and thus would not be able to attach itself to α-MSH_1–12_, since its N-terminus is acetylated. It is also not clear if TMAB or similar labeling could be used to quantitate deacetylated, amidated α-MSH_1–13 _versus acetylated, amidated α-MSH_1–13_. This is a problem for relative comparison of distinct peptides within a sample. Nevertheless, the label quantitative technique works well to compare each peptides paired from different samples. This makes the multiplex peptides quantitation technique described here an ideal follow up technique on discovery made by isotopic label experiment. Multiple forms of the discovered peptides and their associated biology can be investigated using MRM transitions, without the label method restriction and bias.

Another key advantage of this MRM technique is the increase in sensitivity and selectivity, allowing much lower detection limits, than typical MS survey experiments. This helps greatly for the detection of lower abundance neuropeptides. Combined with the increased dynamic range of MS instruments, the coverage of a wide concentration range is possible.

The quantitative technique used in this study can target theoretically most peptides, provided that the MRM transitions encountered in biological samples are identified and the MS instrument is tuned to quantitate them. The use of a standard improves the tuning process greatly, which is important to ensure adequate detection limits. This makes the technique very suitable for quantitative neuropeptidomics, with a special emphasis on monitoring multiple forms of target peptides and/or enzymatic reactions. Compared to RIA, the technique is not restricted by the availability of good antibodies, which can be challenging for protein of low specificity. The technique is also very suitable for analyzing molecules with only small differences, such as PTMs. Because of its sensitivity and specificity, we see our multiplex quantitative peptidomics technique as a potent improvement over existing techniques for neuropeptide studies, such as disease states, inhibitor studies, and others.

Our *in vitro *experiment confirmed PREP digests α-MSH_1–13 _and the appearance of α-MSH_1–12 _in the reaction mix revealed that α-MSH_1–12 _is the product of PREP on α-MSH_1–13_. The reduced level of α-MSH_1–12 _*in vivo *in a PREP deficient pituitaries confirms that the production of α-MSH_1–12 _*in vitro *is due to PREP itself and not some contamination form other enzymes in the rPREP source. Reciprocally, our *in vitro *data strongly suggest that the reduced level of α-MSH_1–12 _in PREP-GT pituitaries is due to reduced PREP levels.

α-MSH_1–12 _has different biological activity than α-MSH_1–13 _because α-MSH_1–12 _does not stimulate action potentials in MC4R expressing neurons. (Wallingford et al. unpublished data). However, it is possible or even probable that α-MSH_1–12 _may activate other receptors that would not be the target of the C-terminus of α-MSH_1–13_. Since it has been shown that the tripeptide KPV, corresponding to the three C-terminus of α-MSH_1–13_, has anti-inflammatory effect, partially through MC1R [21], it is possible that α-MSH_1–12_, lacking a C-terminus valine, can't act as ligand to MC1R. In addition, the cleavage of the terminal valine results in the loss of α-MSH C-terminus amide, which may also affect α-MSH ability to act as a ligand on some melanocortin receptors.

While not significant when comparing the heterozygote to the wild type pituitaries, the ratio of acetylated α-MSH_1–13 _and α-MSH_1–12 _appears correlated to PREP levels. This suggests an additive effect of PREP on the digestion of α-MSH by PREP.

In this experiment, we did not verify the absolute quantity of α-MSH_1–13 _by itself but we hypothesize that in a reduced PREP level condition, α-MSH_1–13 _may have a longer life as its turnover is slowed. We did not observe a significant change in the N-acetyltransferase activity (from an unknown enzyme), which is consistent with a stable absolute level of α-MSH. Our results support previous reports of deacetylated α-MSH_1–13 _in the pituitary [22]. Further leptin, which has been reported to regulate the N-acetyltransferase activity of α-MSH_1–13 _[23], was not found significantly correlated with PREP-GT mice phenotype [13]. This is in support of previously published evidences that pituitary have a sensing mechanism of acetylated α-MSH_1–13 _concentration and they release α-MSH_1–13 _accordingly [24], suggesting a feedback inhibition mechanism that could result in an unchanged absolute quantity of acetylated α-MSH_1–13 _in the PREP-GT mouse. This is in agreement with the lack of obesity phenotype in the analyzed cross [13].

Finally, neuropeptides are signal molecules that can have rapidly changing levels. To assess enzymatic activity on neuropeptides across biological replicates, it is critical to normalize the quantity with endogenous controls. The most suitable endogenous control for enzymatic activity on a substrate is indeed the product of the enzymatic reaction.

## Conclusion

We have established that PREP digests α-MSH_1–13 _and produce α-MSH_1–12 _*in vitro*. The reduced relative level of α-MSH_1–12 _in a reduced PREP level background strongly suggests that α-MSH_1–13 _is a substrate of PREP *in vivo*.

The multiplex targeted quantitative peptidomics techniques demonstrated in this study are a powerful new tool for quantitation of neuropeptides. It opens the possibility for experiments with higher specificity and sensitivity that allows *in vivo *multiplex monitoring of several neuropeptides, under varying conditions.

## Methods

### Identification of MRM transitions using standards

C-amidated α-MSH_1–13 _(deacetylated α-MSH; American Peptide cat# 56-0-24), N-acetylated, C-amidated α-MSH_1–13 _(Bachem cat# H-1075) and N-acetylated α-MSH_1–12_, (custom peptide synthesis from Genscript Corporation; sequence: acetyl-SYSMEHFRWGKP) were directly infused into a Thermo Scientific TSQ Vantage triple quadrupole mass spectrometer to identify the optimum MRM transitions generated by these three compounds. We restricted the analysis to the prevalent charge state of each peptide. MRM transitions were then validated by spiking the three compounds in a biological sample and then analyzed as described in paragraph B below (PREP *in vitro *reaction).

### PREP *in vitro *reaction

We used recombinant 81 Kd PREP (denoted rPREP) from R&D systems (Cat# 4308-SE), and N-acetylated, C-amidated α-MSH_1–13 _(Bachem cat# H-1075). 2 μg α-MSH_1–13 _was incubated in the presence of 50 ng rPREP in a final volume of 50 μL assay buffer (25 nM Tris, 0.25 M NaCl, pH 7.5) for 0, 15, 30 and 60 min at 25°C. Reaction was terminated, by lowering pH to 3.5 by adding HCl 0.7 M. Samples were then desalted using C18 ziptip (Omix C18) following manufacturer instructions.

10 μL of the reaction mixes were analyzed by mass spectrometry using a Thermo Scientific TSQ Vantage with a Michrom Bioresources MDLC and a CTC Pal Autosampler. Peptides were separated using a reverse phase Michrom Magic C18 (200 umx150 mm) column at a flow rate of 2 μL per min. and a 90 min gradient of 2% B to 60% B over 60 minutes (A = 0.1% Formic Acid, B = 100% acetonitrile). Scan width was set at 0.002. Q1 and Q3 peak widths at 0.7.

Peak areas for several MRM transitions for each target peptides were then calculated using the software Xcalibur version 2.0.7. The MS method was targeting, respectively, 5 and 7 MRM transitions for α-MSH_1–13 _and α-MSH_1–12_

### Generation of PREP-GT mice

PREP-GT is a Genetrap mouse generated from the BayGenomics clone RRM213 and back crossed to B6. PREP genetrap presence is confirmed at every generation by genotyping three microsatellite markers (D10Mit148 and D10Mit55 and D10Mit36).

### Mouse maintenance, diet and dissection

An F2 colony was created by breeding PREP^*gt*/*wt *^littermates to produce pups that were heterozygous PREP^*gt*/*wt *^(HT), gene-trap PREP^*gt*/*gt*^(GT), or wild-type PREP^*wt*/*wt *^(WT). Breeding pairs were maintained in a 14 h light/10 h dark cycle, 21 ± 2 (SD) °C temperature, 25% or greater humidity and fed a breeder chow (LabDiet^® ^5015, PMI Nutrition International, St. Louis, MO). Pups were weaned at 3 weeks of age and separated by gender. They were housed 3–5 mice in shoebox cages and placed ad libitum low fat AIN-76A diet (Research Diets, Inc., New Brunswick, NJ) with deionized water. Mice were sacrificed at 120 ± 3 (SD) days of age, after ~15 hours. The pituitaries were dissected and flash frozen.

### Pituitary Western blots

Protein was extracted from whole pituitary samples of each genotype. The pituitary was homogenized on ice in 100 μL NP-40 lysis buffer (150 mM NaCl, 1.0%, NP-40 Tergitol, 50 mM Tris, pH 8.0) supplemented with protease inhibitor cocktail (Roche Cat# 1 697 498 001). The samples were centrifuged at 4°C for ten minutes at 14,000 g. The supernatant was retained and used for Western Blot analysis. Protein concentration was determined through micro-BCA assay (Cat # 23235 Thermo Scientific) and 2.5 μg of total Protein was run on NuPAGE 4–12%Bis-Tris Gel and transferred to GE Nitrocellulose membrane. We performed Ponceau S staining on unblocked membrane for loading control and blocked with 5% milk overnight in a cold room. The membrane was blocked with Primary Antibody (Abcam ab58988) for one hour, washed three times with TBST for ten minutes, then blocked with secondary antibody (Abcam ab6721) for one hour, and finally washed another three times for ten minutes with TBST.

### Mice pituitary multiplex quantitative analysis of 3 forms of α-MSH

The pituitary sample preparation is adapted from methods previously published [25]. Dissected pituitaries were immersed in 100 μL 10 mM HCl, sonicated two or three times for 5 s and incubated at 70°C for 20 min. A 100 μL of 0.2 M phosphate buffer, pH 9.5 was added, and the homogenate was centrifuged at 50,000 g for 40 min at 4°C. The pellet was re-suspended in 200 μl of 0.2 M phosphate buffer, pH 9.5, and centrifuged at 50,000 g for 40 min at 4°C. Supernatant were combined and diluted with 200 μL of 0.4 M phosphate buffer, pH 9.5. The pH was then adjusted to 9.5 with 1 M NaOH. The samples were then run through Strata-X columns per manufacturer instruction (Phenomenex: Strata C18-E (55 um, 70A) 200 mg/3 mL; Cat# 8B-S001-FBJ). After elution with 60% acetonitrile, samples were lyophilized, resuspended in 100 μL 2% acetonitrile, 0.1% formic acid and sonicated in ultra sonic bath for 10 minutes.

Aliquotes containing 2 μg were analyzed with the same equipment and conditions than described above for the *in vitro *reactions mixes, except that we injected 20 μL of the samples and the MS method was targeting, respectively, 4, 5 and 7, MRM transitions for the prevalent charge state of the deacetylated α-MSH_1–13_, the acetylated α-MSH_1–13 _and α-MSH_1–12_.

### Statistical analysis

Statistical significance of quantitation across genotypes was calculated by one tail Student t-test using Microsoft Excel.

## Abbreviations

α-MSH: alpha Melanocyte Stimulating Hormone; MRM: Multiple Reaction Monitoring; PREP-GT: PRolyl EndoPeptidase GeneTrap knock down mouse model; POMC: ProOpioMelanoCortin; RT: Retention Time; PA: Peak Area; TIC: Total Ions Count; WT: Wild Type *Prep*^*wt*/*wt *^genotype; GT: GeneTrap *Prep*^*gt*/*gt *^genotype; RIA: RadioImmunoAssay; PTM: Post-Translational Modifications; TMAB: trimethylammoniumbutyrate.

## Competing interests

The authors declare that they have no competing interests.

## Authors' contributions

BP: experiment design and implementation, data analysis, manuscript writing.

GME: sample preparation and manuscript editing ARM: sample preparation RJA and BSP: MS experiments and manuscript editing.

CHW: advise on experiment design, manuscript editing and funding.

## Supplementary Material

Additional file 1 **PREP *in vitro *protease activity on α-MSH_1–13_produces α-MSH_1–12_**. Panel A shows the TIC for each α-MSH_1–12 _MRM transitions (seven left plots) at the beginning of the reaction (T0) and panel B (seven right plots) after one hour incubation (T60). The peak areas after 1 hour of incubation (T60) are about 500 fold of those without incubation (T0), for equivalent injected reaction mix amount. Click here for file

Additional file 2 **Detection and quantitation of 3 α-MSH forms using MRM transition methods in pituitary of wild type mouse**. The TIC peaks of the (+3) charge state for the 4, 5 and 7 MRM transitions of respectively, deacetylated α-MSH_1–13_, acetylated α-MSH_1–13 _and α-MSH_1–12 _are shown respectively, in **panel A, B and C**. Peaks are labeled with the retention time (RT) and the peak area (PA) as calculated with the MS software Xcalibur. The three panels come from a single multiplexed experiment on the same pituitary peptide sample. **Panel A **shows the 4 MRM transitions of deacetylated α-MSH_1–13_. **Panel B**, the 5 MRM transitions of acetylated, α-MSH_1–13 _and **Panel C **the seven transitions for α-MSH_1–12_. Quantitation of a compound is calculated by summing the peak areas of all the MRM transitions of a given compound. Click here for file
