# Supplementary figures and images for: In vivo multiplex quantitative analysis of 3 forms of alpha melanocyte stimulating hormone in pituitary of prolyl endopeptidase deficient mice
Source: Mol Brain. 2009 Jun 2;2:14. doi: 10.1186/1756-6606-2-14 (PMC2698928; doi:10.1186/1756-6606-2-14)

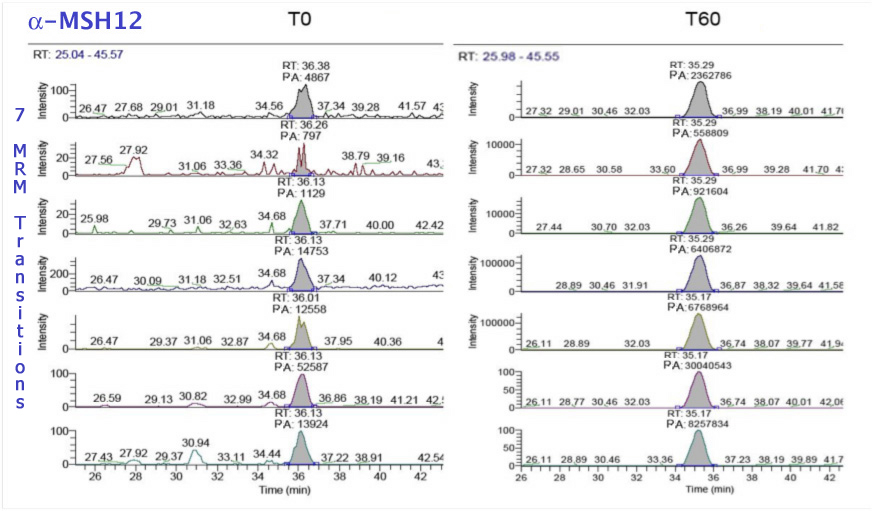

Supplement: Additional file 1 — PREP in vitro protease activity on α-MSH1–13produces α-MSH1–12. Panel A shows the TIC for each α-MSH1–12 MRM transitions (seven left plots) at the beginning of the reaction (T0) and panel B (seven right plots) after one hour incubation (T60). The peak areas after 1 hour of incubation (T60) are about 500 fold of those without incubation (T0), for equivalent injected reaction mix amount. [file 1756-6606-2-14-S1.jpeg]

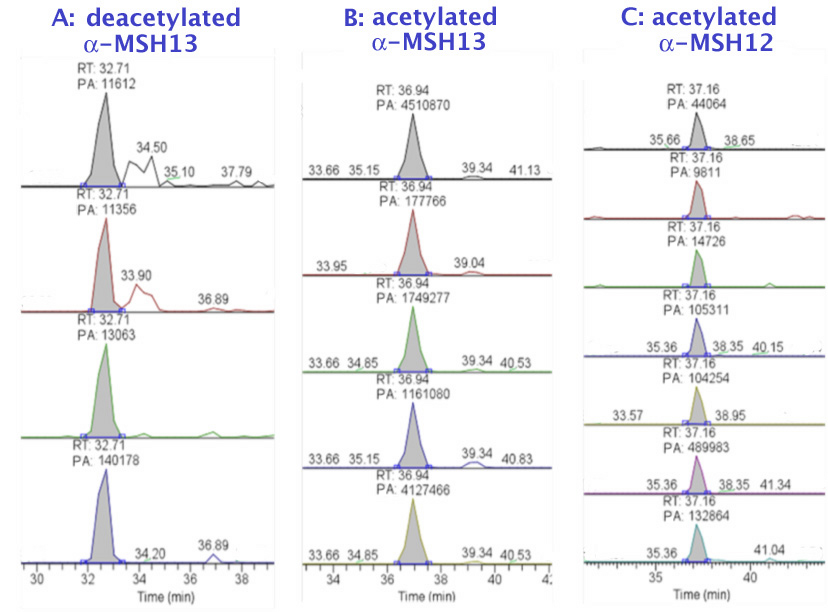

Supplement: Additional file 2 — Detection and quantitation of 3 α-MSH forms using MRM transition methods in pituitary of wild type mouse. The TIC peaks of the (+3) charge state for the 4, 5 and 7 MRM transitions of respectively, deacetylated α-MSH1–13, acetylated α-MSH1–13 and α-MSH1–12 are shown respectively, in panel A, B and C. Peaks are labeled with the retention time (RT) and the peak area (PA) as calculated with the MS software Xcalibur. The three panels come from a single multiplexed experiment on the same pituitary peptide sample. Panel A shows the 4 MRM transitions of deacetylated α-MSH1–13. Panel B, the 5 MRM transitions of acetylated, α-MSH1–13 and Panel C the seven transitions for α-MSH1–12. Quantitation of a compound is calculated by summing the peak areas of all the MRM transitions of a given compound. [file 1756-6606-2-14-S2.jpeg]
